# Cases of an Affection Characterised by Involuntary Motions of the Limbs While Walking, Simulating Drunkenness, and Cured by Opium

**Published:** 1832-06

**Authors:** 


### AFFECTION SIMULATING DRUNKENNESS.

*Cases of an Affection characterised by involuntary Motions of the Limbs while Walking, simulating Drunkenness, and cured by Opium.* Reported from the Military Hospital of Strasbourg, by Dr. RENNES.*

CASE I. N—, a musician of the 47th regiment of the Line, aged twenty-five, of a sero-sanguineous temperament and middle height, after a syphilitic affection contracted in 1822, had repeatedly suffered from exostosis of the upper part of the cranium and of the tibiæ. These nodes, being at first treated by mercurials, and afterwards on the antiphlogistic regimen, slowly disappeared, so that at the end of two years no trace of them remained; when he presented himself at the Strasbourg hospital, in the month of September, 1824, with acute bronchitis, complicated by some asthmatic symptoms. Going out cured in October, this man, who was much addicted to spirituous liquors, abandoned himself to drunkenness; and in December he returned in the following state:

The pulmonary symptoms had completely left him; but, for the last fortnight, the motions of his limbs had become less and less under the command of his will. When he wished to walk forwards, he deviated from the right line, constrained

* Archives Gen. de Med.

by a power superior to volition, sometimes moving to the right, and sometimes to the left, but particularly in the latter direction. "The street," he says, "is not wide enough for him." His appetite good, and the other functions not deranged, only they appeared enfeebled, and there was an observable half-intermittence of the pulse at every seven beats. This man having been minutely examined for several days, the physicians of the hospital, of which number I am one, agreed in consultation to try the effect of opium, on account of the resemblance between this disease and delirium tremens, and likewise from the circumstance of the previous syphilitic taint. The dose began with was one grain of the extract, and this was increased daily by the addition of another grain. On the fourth day, the pulse, always slow, lost the intermittence. By the 25th of December, the opium was given to the etxent of nine grains per diem, and the patient slept from six o'clock in the evening to eight the following morning, but remained perfectly awake during the rest of the day, and walked with much greater regularity. The opium was omitted for twenty-four hours; but on the 26th was continued, ten grains being then given. On the 27th, an exostosis was observed in front of one of the tibiæ; on the 28th, two more appeared on the right arm; and the opium that day administered amounted to twelve grains. This dose was not increased, as the patient could regulate his walking, and then the opium was decreased two grains daily.

By the the 4th of January, 1825, the soldier whose case has been now recorded, having recovered his strength, left the hospital, and marched away with his regiment, which was ordered to other quarters.

Case II. Louis ***, short, stout, and thick set, presenting all the characteristics of the lymphatic temperament, was admitted into the Strasbourg Military Hospital, on the 9th of August, 1828, on account of a catarrh, with which he had been affected for upwards of three weeks, and which had previously been entirely neglected; the cough was violent and constant, accompanied with much weakness, loss of appetite, frequent pulse, and heat of skin, especially increased towards evening and during the night; expectoration easy; sputa white, thick, and glairy. This attack was at first treated by emollient pectoral medicines, mucilaginous drinks, and milk diet. These means not having been followed by any relief, twenty leeches were applied on the third day to the upper part of the sternum, after which the cough and fever immediately lessened, although the difficulty of breathing increased and the expectoration was abundant. On the morning of the 19th a blister was applied to the chest, from which great benefit ensued. A mild laxative was afterwards given, and on the 1st of September the man was convalescent.

I was astonished, at my visit on the morning of the 6th, to find Louis worse than on the preceding days, restless, and suffering with violent headach, seated at the superior anterior part of the frontal bone; at the same time the pulse had become hard, sharp, and frequent, and the skin hot: the face continued, as usual, pale; tongue moist and white; thirst slight; appetite gone; otherwise there existed not any symptom of bronchitis. Two days' abstinence, assisted by mustard pediluvia, relieved him greatly; and on the 13th a slight weight in the frontal region alone remained, when the patient told us that three or four days previously, when walking in the airing ground of the hospital, his companions laughed at him for the awkwardness of his gait, and the continual zigzags that he made, sometimes going to the right, sometimes to the left, without the possibility of walking in a more regular manner.

This curious phenomenon, which I had witnessed in another individual four years ago, struck me the more forcibly this time, inasmuch as it occurred under nearly similar circumstances, and as I had just been reading of an analogous case recorded in the "Archives générales de Médecine," tome xvii., by Dr. Dufour, in which a similar state had at first been mistaken for real intoxication, but which was attended with paralysis and terminated in death.

This patient drew my attention in an especial manner: I retraced with care all the circumstances he recollected, and learned from him, 1st, that being little inclined to sexual pleasures, he indulged the more freely in the use of spirits, still without being an habitual drunkard, as his means would not permit him; 2d, that he had been in the service for eight years, and had never been attacked with any other disorder. I reconsidered the nature and seat of the pain in the head, fixed as it had been to the upper part of the forehead, which had been relieved for some days, but which left a sensation of cramp and heaviness on the part. Afterwards I made him walk in my presence, and observed, in fact, that he could not pursue a straight direction; that, in spite of all his efforts, and his attention fully directed to the point, he was forced involuntarily sometimes to the right, and sometimes to the left, but more especially to the left. At this period the pulse were natural and tongue clean, appetite good and bowels regular, hearing acute, sight clear, and the pupil contractile. However, when the man had crossed the room several times, his sight was for a short time obscure, he became giddy, and was compelled to sit down to avoid falling. His ideas were not confused, but his countenance had something of the vacancy of drunkenness. The mildness and tractability of this patient were remarkable, and nothing could give rise to the suspicion that he had any intention to deceive.

The first day that these symptoms were observed, I made it my business to watch him, and only prescribed a mustard pediluvium. On the 14th, I ordered five leeches to be applied behind each ear, and on the morrow a blister to the nape of the neck. On the 16th, the headach greatly subsided, and he told us that he could walk much better; but, on being put to the proof, I found that there was little alteration in this respect, and he still exhibited all the appearance of intoxication: he could scarcely raise his hand to his hat, to give the military salute, and his speech was slow and slightly embarrassed. The 17th and 18th passed without any particular change; he only complained of want of sleep. This last circumstance, and the recollection of the former case led me to try the extract of opium: I gave him one grain every night for four times. On the 24th, he walked more firmly, and the watchfulness had entirely ceased. On the 25th, he complained of a sensation of heat in his head, which induced me to suspend the use of the opium for several days; and fever having supervened during the night, I applied, on the 26th, ten leeches to the temples, and on the following day twelve more on the occiput; and the symptoms were partially relieved by this treatment.

By the 1st of October, the unsteadiness of his carriage was much reduced, but the irregular fever with which he had for some days been affected assumed the intermittent type. Sulphate of quinine was in consequence combined with opium, six grains of each being exhibited every day; and on the 5th, although weakened by the ague, he walked with a much greater degree of firmness. The last attack was on the 10th, and afterwards he gained strength rapidly. His hair in great part fell off; but his appearance was improved, his skin had a more healthy hue, and his appetite increased daily.

On the 13th of October, he was submitted to another examination, when he appeared still firmer on his legs, although he said he felt a slight tendency toward tbe left side. On the 18th he was completely well, he walked straight forwards, a little hesitation alone remaining, the consequence of want of power or of confidence, which time would speedily remove. Leaving the Strasbourg hospital at this period, I could not follow the history further.

What is the nature of this complaint? Until facts are multiplied, and pathological anatomy has thrown light upon the question, it is one that will be difficult to answer. With regard to its seat, that cannot be elsewhere than in the brain, and the locality of the pain seems to indicate the part more especially affected. The most notable points are these: viz. the resemblance of the symptoms to the phenomena of drunkenness, and the preexistence in both cases of acute bronchitis with violent fits of coughing, the dyspnœa, the habits of intoxication in both men, and finally the success of opium in the cure of both the patients. It is always necessary to make a certain number of observations, and to collect a certain number of cases of a similar description, before we attempt to trace the history of a disease such as this, which I have nowhere seen described, and which I believe to be very rare, for among the vast number of sick that I am continually attending I have met with these two cases only.

---

